# Overweight and Obesity in Children and Adolescents

**DOI:** 10.4274/jcrpe.1471

**Published:** 2014-09-05

**Authors:** Neslihan Koyuncuoğlu Güngör

**Affiliations:** 1 Louisiana State University Health Sciences Center-Shreveport, Department of Pediatric Endocrinology, Shreveport, LA, USA

**Keywords:** overweight, obesity, Pediatric, prevention, Diabetes, weight management, dyslipidemia, type 2 diabetes mellitus

## Abstract

Obesity among children, adolescents and adults has emerged as one of the most serious public health concerns in the 21st century. The worldwide prevalence of childhood obesity has increased remarkably over the past 3 decades. The growing prevalence of childhood obesity has also led to appearance of obesity-related comorbid disease entities at an early age. Childhood obesity can adversely affect nearly every organ system and often causes serious consequences, including hypertension, dyslipidemia, insulin resistance, dysglycemia, fatty liver disease and psychosocial complications. It is also a major contributor to increasing healthcare expenditures. For all these reasons, it is important to prevent childhood obesity as well as to identify overweight and obese children at an early stage so they can begin treatment and attain and maintain a healthy weight. At present, pharmacotherapy options for treatment of pediatric obesity are very limited. Therefore, establishing a comprehensive management program that emphasizes appropriate nutrition, exercise and behavioral modification is crucial. The physician’s role should expand beyond the clinical setting to the community to serve as a role model and to advocate for prevention and early treatment of obesity.

## INTRODUCTION

Obesity among children, adolescents and adults has emerged as one of the most serious public health concerns in the 21st century. The worldwide prevalence of childhood obesity has increased strikingly over the past 3 decades (1). Obesity is a multifactorial condition and has also been described as a phenotype of numerous pathologies (1,2). This review intends to analyze the current medical literature pertaining to overweight and obesity in the pediatric age group and highlight the clinically pertinent practical points. The definition, prevalence, epidemiology and comorbidities of pediatric obesity will be reviewed. Clinical and laboratory evaluation and treatment issues will be discussed. Given the extensive nature of the reviewed topic, the reader will be referred to recent articles on selected areas, as appropriate, for a more in-depth review.

**Definition**

Obesity is characterized by an excess of body fat or adiposity. Obesity is most often defined by the body mass index (BMI), a mathematical formula of weight-for-height index. BMI is measured by dividing the body weight in kilograms to height in meters squared (kg/m2). BMI has a high correlation with adiposity and it also correlates well with excess weight at the population level. It is important to note that the calculated BMI figure can sometimes be inaccurate because it does not quantify total body adiposity, does not distinguish between fat and muscle, nor does it predict body fat distribution. Therefore, it may overestimate adiposity in a child with increased muscle mass, as may be the case in an athletic child and underestimate adiposity in a child with reduced muscle mass, such as a sedentary child. On a population level, however, BMI does seem to track trends in adiposity as opposed to muscularity and those individuals with large muscle mass with resulting high BMI values are easily distinguishable from those with large amounts of adipose tissue (3,4,5). The World Health Organization (WHO) categorizes adults with a BMI of 25 to 30 as overweight, whereas obesity is classified according to stages or grades - Grade 1: BMI 30.0-34.9, Grade 2: BMI 35.0-39.9 and Grade 3: BMI ≥40.0 (4). Grade 3 obesity was formerly known as morbid obesity, but the term was appropriately changed for several reasons: morbidity may not occur at BMI levels higher than 40 but certainly can be found at BMI levels lower than 40 (4). In the pediatric age group, gender-specific BMI-for-age percentile curves are used to define overweight and obesity. Children and adolescents with a BMI over the 85th but less than the 95th percentile for age and gender are considered overweight and those with a BMI greater than the 95th percentile are considered obese. Children and adolescents with a BMI greater than the 99th percentile are considered severely obese (2,3,6). The International Obesity Task Force has developed an international standard growth chart which enables comparison of prevalence globally (7). However, many countries have continued to use country-specific growth charts. In the United States, the gender-specific CDC Growth Charts, released in May 2000, are used to evaluate BMI for children 2 to 20 years of age (6).

**Etiology, Determinants and Risk Factors**

**Figure 1:**
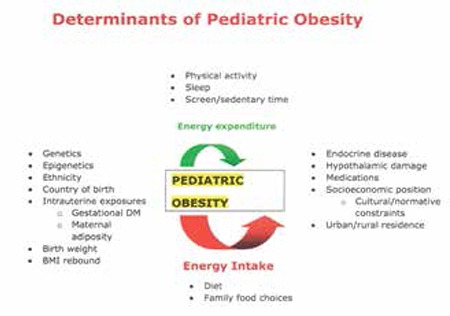
Determinants of pediatric obesity (1,2,8,9,10)

Obesity is a complex, multifactorial condition affected by genetic and non-genetic factors (1,2). Figure 1 outlines the determinants of pediatric obesity. In children and adolescents, the overweight state is generally caused by a lack of physical activity, unhealthy eating patterns resulting in excess energy intake, or a combination of the two resulting in energy excess. Pediatric obesity is also a multifactorial condition which is a resultant of genetic and non-genetic factors and the complex interactions among these. Genetics and social factors (socio-economic status, race/ethnicity, media and marketing and the physical environment) also influence energy consumption and expenditure (8). Obesity seems to be the result of a complex interplay between the environment and the body’s predisposition to obesity based on genetics and epigenetic programming (9). To date, research has been unable to isolate the effects of a single factor due to the co-linearity of the variables as well as research constraints.

Specific causes for the increase in prevalence of childhood obesity are not clear and establishing causality is difficult since longitudinal research in this area is limited. The heritability of body weight is high and genetic variation plays a major role in determining the interindividual differences in susceptibility or resistance to the obesogenic environment (10). Appetite regulation and energy homeostasis depend on a large number of hormones many of which are secreted by the gastrointestinal tract (9). Ghrelin is currently the only known appetite-stimulating (orexigenic) gut hormone, secreted by the oxyntic glands of the stomach. Ghrelin levels rise shortly before mealtimes. The other gut hormones identified to date are anorexigenic (decrease appetite and food intake). These include: peptide tyrosine tyrosine (PYY), pancreatic polypeptide, oxyntomodulin, amylin, glucagon, glucagon-like peptide-1 (GLP-1) and GLP-2. For example, PYY acts as a satiety signal. The levels of PYY rise within 15 minutes after food intake, resulting in reduced food intake (11). The gastrointestinal tract is the body’s largest endocrine organ producing hormones that have important sensing and signaling roles in the regulation of energy homeostasis (12). There are several determinants or risk factors for development of pediatric overweight or obesity. These can be categorized as follows (1).

**Genetic Variation**

There are rare single gene defects in which obesity is the specific abnormality. Abnormalities in the leptin signaling pathway is an example (1,13). Onset of marked obesity during early infancy raises the suspicion for genetic mutations in the leptin signaling pathway or for melanocortin-4 receptor abnormalities. These are exceedingly rare conditions. The most common single gene defect causing pediatric obesity is melanocortin-4 receptor defects which comprise 5%-6% of early-onset pediatric obesity (10).

Obesity is a manifestation of several genetic syndromes such as Prader-Willi syndrome, Bardet-Biedl syndrome, Alstrom syndrome and WAGR syndrome (1,10,13). For example, Prader-Willi syndrome is a clinical entity characterized by hypotonia, mental retardation, short stature, hypogonadism, hyperphagia and obesity. It is caused by lack of expression of genes on chromosome 15q11-13, which are imprinted such that they are only expressed on the paternally inherited copy of the chromosome (10).

Common genetic variants associated with greater adiposity and obesity have also been identified by genome-wide association studies (1,14).

**Epigenetics**

Involves the mechanism through which in utero factors can produce heritable changes in adiposity, which has been suggested to be due to DNA methylation or histone modification of DNA in gene regulatory regions. Research data on humans is scanty and integrated research on larger cohorts are needed (1,15).

**Endocrine Disease**

Hypothyroidism (primary or central), growth hormone deficiency or resistance and cortisol excess are classical examples of endocrine conditions leading to obesity.

Polycystic ovarian syndrome (PCOS) is considered a consequence of but also a possible contributor to obesity. Pseudohypoparathyroidism (caused by Gsα inactivating mutation) is also associated with obesity (1). Albright hereditary osteodystrophy (AHO) is an autosomal dominant disorder resulting from germline mutations in GNAS1 that decrease expression or function of Gsalpha (Gsα) protein. Maternal transmission of GNAS1 mutations leads to AHO (characterized by short stature, obesity, skeletal defects and impaired olfaction) plus resistance to several hormones (e.g. parathyroid hormone) that activate Gsα in their target tissues. Paternal transmission leads only to the AHO phenotype (16).

**Central Nervous System Pathology**

Congenital or acquired hypothalamic abnormalities have been associated with a severe form of obesity in children and adolescents (1,17). Hypothalamic injury from acquired structural damage due to infiltrative disease, tumor or their treatment aftereffects frequently results in the development of an obesity syndrome characterized by a rapid, unrelenting weight gain that may be accompanied by severe hyperphagia. Weight gain occurs from the disruption of the normal homeostatic functioning of the hypothalamic centers responsible for controlling satiety and hunger and regulating energy balance with resulting hyperphagia, autonomic imbalance, reduction of energy expenditure and hyperinsulinemia (18).

**Intrauterine Exposures**

a) Intrauterine exposure to gestational diabetes: In populations at high risk of obesity and diabetes (e.g. Pima Indians) exposure to gestational diabetes is associated with increased risk of childhood and early adult obesity in the offspring (1,19).

b) Intrauterine exposure to greater maternal adiposity: Some studies have shown that extreme variation in maternal adiposity (for example, achieved through bariatric surgery) affects offspring obesity. The prevalence of overweight and obesity in children of mothers with large voluntary postsurgical weight loss was similar to that in the general population, with no increase in underweight (1,20).

c) Birth weight: Higher birth weight is associated with increased fat as well as increased lean mass in the offspring. Small for gestational age babies who exhibit catch-up growth may be at risk for childhood obesity (21,22). 

**BMI Rebound**

BMI normally decreases after birth, until 5 1/2 years of age when the child experiences an adiposity rebound. The beginning of the post-infancy rise in the BMI has been termed the adiposity rebound and several studies have found that an early rebound increases the risk for overweight in adulthood. Although an early BMI rebound was related to higher levels of relative weight in adulthood, this association was not independent of childhood BMI levels (23).

**Diet**

Breast feeding is unlikely to be causally protective of childhood obesity. Available evidence suggests that high energy intake in early infancy and high consumption of sweetened drinks in childhood is prospectively associated with childhood obesity risk (24,25,26).

**Energy Expenditure**

Low levels of physical activity, greater hours of TV/other screen time are associated with childhood obesity risk.

**Sleep**

Shorter sleep duration in infancy and childhood is associated with childhood obesity risk (27).

**Infection **

The potential role of microbial infections (e.g.Adenovirus Ad-36 infection) and composition of gut flora (e.g. ratio of Firmicutes to Bacteriodetes species) have been suggested to be associated with development of obesity. Epidemiological evidence is lacking at this time (28,29).

**Iatrogenic**

The following have been associated with greater weight gain in children and adolescents (1,17): 

a) Cranial irradiation or surgery causing hypothalamic damage, 

b) Psychotropic medication (e.g. olanzapine and risperidone), 

c) Chemotherapeutics (treatment of acute lymphocytic leukemia even without cranial irradiation), 

d) Hormonal contraception (e.g. depot medroxyprogesterone acetate).

**Ethnic Origin**

Some ethnic groups (e.g. Hispanic and South Asian) appear to have a tendency for overweight (30,31).

**Country of Birth**

Children from low- and middle-income countries tend to be stunted and underweight but with sufficient nutrition gain healthy weight and with overnutrition are prone to obesity (32).

**Urban Versus Rural Area of Residence**

Children in urban areas are more likely to be obese than those in rural areas in many countries including high- and low/middle-income countries (33).

**Socioeconomic Level**

Contemporary populations of children in high-income countries show higher rates of obesity in their lowest socioeconomic groups. No socioeconomical adiposity differentials in children born before 1950’s-1960’s has been reported in high-income countries (34).

**Prevalence and Epidemiology**

The worldwide prevalence of childhood obesity has increased greatly over the past 3 decades (1). In the United States (US), the incidence of pediatric obesity has increased from less than 5% to approximately 20% in the past 30 years (35). Prevalence estimates of obesity are derived from surveys or population studies. In the US, the National Center for Health Statistics of the CDC program provides national estimates of overweight for adults, adolescents and children. In 2007-2008, 16.8% of US children and adolescents had a BMI greater than or equal to the 95th percentile on the BMI-for-age charts and were considered obese. Although significant increases in obesity prevalence were seen in both sexes of children and adolescents during the 1980s and 1990s, between 1999-2000 and 2007-2008, significant increases were seen only at the highest cut point of BMI, corresponding to the 97th percentile, in 6- through 19-year-old males. No change at any cut point was seen in females (36).

**Global Prevalence and Trends of Pediatric Overweight/Obesity**

Based on the analysis of 450 nationally representative cross-sectional surveys from 144 countries in a 2010 report, 43 million preschool children under age 5 (35 million in developing countries) were estimated to be overweight and obese and 92 million were at risk of overweight (37). Overweight and obesity were defined as the proportion of preschool children with values >2 SD and >3 SD respectively from the WHO growth standard median. Being “at risk of overweight” was defined as the proportion with values >1 and ≤2 SD. The worldwide prevalence of childhood overweight and obesity increased from 4.2% in 1990 to 6.7% in 2010. The estimated prevalence of overweight in 2010 was 8.5% in Africa and 4.9% in Asia. A WHO report drawing data from the Health Behaviour in School-Aged Children (HBSC) 2005/2006 survey covering 36 countries in the WHO European Region depicted that the prevalence of overweight/obesity in 11- and 13-year olds ranges from 5% to more than 25% in some countries (38). Comparison of data from the 2001/2002 and 2005/2006 HBSC surveys indicated that the situation was not improving. Other reports point to similar trends in global prevalence of childhood obesity.

**Comorbidities and Complications**

Obesity is a proinflammatory state that increases the risk of several chronic diseases encompassing hypertension, dyslipidemia, diabetes, cardiovascular disease, asthma, sleep apnea, osteoarthritis and several cancers in adults (2,4). With the increasing prevalence of pediatric obesity, clinicians have started to identify many of the same chronic illnesses and risk factors that are seen in adults in pediatric age groups. Childhood obesity can adversely affect nearly every organ system and often has serious consequences, including hypertension, dyslipidemia, insulin resistance, prediabetes, type 2 diabetes mellitus (T2DM), fatty liver disease and psychosocial complications (1,2,4,5,39,40). In general, comorbidities of pediatric obesity may be grouped under physical and psychosocial categories.

It is also notable that body fat and the specific depot where the adipose tissue is stored (visceral vs. subcutaneous) can contribute to cardiometabolic health risks in children and adolescents. There are multiple studies indicating that visceral adipose tissue in particular is predictive of comorbidities such as insulin resistance, hypertension and hepatic steatosis (41). 

The prevalence of obesity-related comorbidities has been on the rise parallel to the increasing prevalence of pediatric obesity. These comorbidities have been listed in (Table 1).

Insulin resistance is the common denominator for many of the metabolic and cardiovascular complications of obesity. Hyperinsulinemia is the most common biochemical abnormality seen in obesity. Insulin resistance with compensatory hyperinsulinemia is the initial step in T2DM pathogenesis. The subsequent step is impaired early insulin secretion, leading to post-prandial and later, fasting hyperglycemia, at which time clinical diabetes becomes manifest (40,42,43). Impaired glucose tolerance is a relatively common condition in obese children and adolescents, with a reported prevalence ranging from 15% to greater than 20% (44,45,46). Owing to the secular trends of pediatric obesity, since late 1980s, T2DM has been transformed from a disease historically known to affect only adults, to a serious pediatric public health problem affecting children as young as 6 years old (40,47). The SEARCH for Diabetes in Youth Study, which is the largest surveillance effort of diabetes in youth conducted in the US, has identified significantly increased rates of T2DM in adolescents of minority origin of ages 10 to 19 years. Among these adolescents, the proportion of T2DM ranged from 6% (0.19 cases per 1000 youth for non-Hispanic white youth) to 76% (1.74 cases per 1000 youth for American Indian youth). The reported prevalence figures were 22% in Mexican American youths, 33% in African American youths and 40% in Asian/Pacific Islander youths (48). Prediabetes (impaired fasting glucose and/or impaired glucose tolerance) requires particular attention from a secondary prevention perspective to hinder conversion to T2DM.

The observation that certain metabolic disturbances appeared to cluster together has been noted since the beginning of the past century. Insulin resistance has been proposed as a common etiologic factor for the group of metabolic disturbances collectively referred to syndrome X or the metabolic syndrome (MetS). Current definitions of MetS include the following key characteristics: Hyperinsulinemia or insulin resistance, dyslipidemia, hypertension and obesity, with a particular emphasis on central adiposity. Pediatric MetS represents a cluster of risk factors associated with cardiovascular disease, with features that include insulin resistance, obesity, hyperlipidemia and hypertension. Currently, there are no consensus definitions to define the MetS in youth; however, identification of children at risk for developing MetS remains an important task because of the presence of multiple cardiovascular risks and the evidence that the clustering of these conditions persists in adulthood (49). It has been documented that early stages of the atherosclerotic process are detectable in obese children. Endothelial dysfunction represents the key early step in the development of atherosclerosis (50). Intima-media thickness (IMT) of the peripheral arterial vessels is a surrogate marker for atherosclerosis and increased IMT has been documented in obese children and adolescents (51). The Bogalusa Heart study is a landmark study which demonstrated that cardiovascular risk factors present in childhood are predictive of coronary artery disease in adulthood (52). Among the risk factors, low-density lipoprotein (LDL) cholesterol and BMI measured in childhood were found to predict IMT in young adults. In a recent report on the follow-up of childhood participants of the Bogalusa Heart Study as adults, children who have metabolically healthy overweight/obesity were noted to have favorable cardiometabolic profiles in adulthood. Metabolically healthy overweight/obesity was defined as being in the top BMI quartile, while LDL cholesterol, triglycerides, mean arterial pressure and glucose were in the bottom 3 quartiles and high-density lipoprotein (HDL) cholesterol was in the top 3 quartiles. Despite markedly increased obesity in childhood and in adulthood, these metabolically healthy children and adults showed a cardiometabolic profile generally comparable to that of non-overweight/-obese children. There was no difference in carotid IMT in adulthood between metabolically healthy children and non-overweight/-obese children (53).

PCOS, characterized by hyperandrogenism and oligo/anovulation in adolescents, is listed as a cause of infertility (54). A recent Endocrine Society Clinical Practice guideline recommends using the Rotterdam criteria (presence of two of the following criteria: androgen excess, ovulatory dysfunction, polycystic ovaries) for diagnosing PCOS. Establishing a diagnosis of PCOS is problematic in adolescents. Hyperandrogenism is central to the presentation in adolescents. Evaluation of adolescents with PCOS should exclude alternate androgen-excess disorders.

Obesity causes changes in other hormonal systems. Age of onset of puberty continues to decrease, particularly in African Americans. This has been attributed, in part, to overnutrition and increased BMI values in this population (55). Excessive aromatization of androgens to estrogens by peripheral adipose tissue may promote gynecomastia in males. Obstructive sleep apnea is among the pulmonary complications of obesity and the hypercapnia associated with this can suppress hypothalamic gonadotropin-releasing hormone function and lead to delayed puberty (56). Obesity accelerates statural growth and causes advancement of the bone age.

Thyroid function aberrations are frequently encountered as most physicians screen overweight/obese children for thyroid function (57). Elevated thyroid-stimulating hormone (TSH) concentrations in association with normal or slightly elevated free thyroxine (T4) and/or free triiodothyronine (T3) levels have been consistently found in obese subjects, but the mechanisms underlying these thyroid hormonal changes are still unclear. Whether higher TSH in childhood obesity is adaptive (increasing metabolic rate in an attempt to reduce further weight gain) or indicates subclinical hypothyroidism or resistance and thereby contributes to lipid and/or glucose dysmetabolism, remains controversial. It is also important to keep in mind that most of the weight gain in hypothyroid individuals is due to accumulation of salt and water, so hypothyroidism rarely causes substantial weight gain.

Cardiovascular comorbidities include hypertension, dyslipidemia and risks for adult coronary heart disease as discussed above. Cardiovascular disease is the leading cause of adult mortality and morbidity. Longitudinal epidemiologic studies have demonstrated that risk factors in childhood, such as obesity and dyslipidemia, are predictors of adult cardiovascular disease. The prevalence of clinically recognized hypertension and dyslipidemia were found to increase 8.5-fold and 3.1-8.3-fold, respectively, in overweight versus lean adolescents in the Bogalusa Heart Study. Excess weight in adolescence persists into young adulthood and has a strong adverse impact on multiple cardiovascular risk factors, indicating the importance of primary prevention early in life (58).

Gastrointestinal comorbidities include nonalcoholic fatty liver disease (NAFLD), nonalcoholic steatohepatitis (NASH), cirrhosis and cholelithiasis (9). NAFLD has become the most common cause of chronic liver disease in children in the US, parallel to the increasing frequency of obesity (9,59). NAFLD represents the fatty infiltration of the liver in the absence of alcohol consumption. NAFLD encompasses a range of severity from bland steatosis to NASH that may ultimately result in advanced fibrosis, cirrhosis and hepatocellular carcinoma. Because NAFLD is usually asymptomatic, screening is required for detection. Non-invasive evaluation can be used to make the diagnosis of NAFLD; detection of markers of liver injury (elevated liver enzymes) or of fibrosis and/or fatty infiltration on ultrasound or magnetic resonance imaging. The clinician must be cognizant of the fact that these diagnostic methods are suboptimal in sensitivity and specificity for NAFLD and other causes of liver disease must be ruled out. The differential diagnosis of elevated transaminases in overweight/obese children should include hepatitis B, hepatitis C, autoimmune hepatitis, α1-antitrypsin deficiency and in older children, Wilson disease. NASH is the progressive form of NAFLD and may progress to cirrhosis (59). Liver biopsy remains the standard criterion for the staging and grading of NAFLD. Insulin resistance is frequently identified in both adults and children with NAFLD (60).

Pulmonary comorbidities include obstructive sleep apnea and obesity hypoventilation syndrome (2,9,61,62). Obese children are up to six times more likely than lean children to have obstructive sleep apnea. Obstructive sleep apnea syndrome is a disorder of breathing during sleep characterized by prolonged partial upper airway obstruction and/or intermittent complete obstruction (obstructive apnea) that disrupts normal ventilation during sleep and also distorts normal sleep patterns. Symptoms include habitual (nightly) snoring (often with intermittent pauses, snorts, or gasps), disturbed sleep and daytime neurobehavioral problems. Daytime sleepiness may occur (62). Obstructive sleep apnea is independently related to the development of hypertension, cardiovascular disease, behavioral disorders and poor school performance in children (63). The prevalence of asthma is also increased in obese children.

Orthopedic complications encompass slipped capital femoral epiphysis (SCFE), genu valga, tibia vara (Blount disease) and fractures. SCFE presents with hip/knee pain and decreased internal rotation of the hip. Blount disease presents with pain at the medial aspect of the knee. The clinician should have a low threshold for suspicion of SCFE in the obese child with knee, thigh, or hip pain with or without antecedent trauma. Overweight children have a higher incidence of fractures (64).

Neurologic complications include idiopathic intracranial hypertension, a disorder which typically presents with headache and blurred vision and is diagnosed by presence of papilledema and elevated intracranial pressure in the absence of infectious, vascular, or structural causes. It can lead to blindness in up to 10% of patients, particularly if it is not recognized or treated promptly (65). The prevalence of pseudotumor cerebri also increases 15-fold with increasing BMI. The risk of idiopathic intracranial hypertension is not related to the degree of obesity and is increased even in individuals who are just 10% above the ideal body weight (65,66).

**Figure 2:**
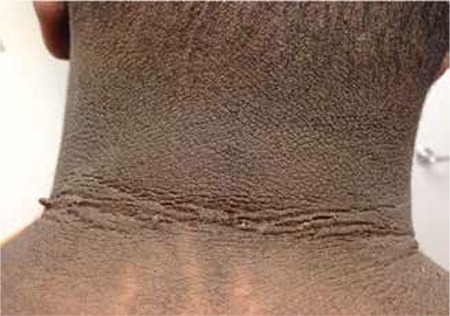
15-year-old African-American male with obesity and severeacanthosis nigricans with skin tags (Photo taken in the author’s clinicafter informed consent)

As to dermatologic complications, obesity alters the skin barrier, can induce skin manifestations and worsens existing skin diseases like psoriasis. Cutaneous manifestations of obesity include acanthosis nigricans (formerly named pseudo-acanthosis nigricans), fibroma pendulans (skin tags, fibroepithelial polyps) and striae distensae (67). Acanthosis nigricans is a dermatologic condition which is associated with obesity, T2DM and insulin resistance (40,68) (Figure 2). Acanthosis nigricans is a velvety thickening of the epidermis that primarily affects the axillae, posterior neck fold, flexor skin surfaces and umbilicus. Clinically, the lesions appear as dark-brown thickened plaques. Histologically, it is characterized by the proliferation of epidermal keratinocytes and fibroblasts. Acanthosis nigricans is an important cutaneous marker of insulin resistance that is more commonly being diagnosed in obese children and adolescents worldwide. Treatment involves management of the underlying disorder (68). Obesity is also associated with hyperandrogenism in women and girls, promoting acne vulgaris, hirsutism and androgenic alopecia. In addition, there is a pathogenic association between obesity and psoriasis: the release of pro-inflammatory factors from fat tissue results in the worsening of psoriasis; an association between the severity of psoriasis and the BMI level has been shown. Obesity promotes skin infections like erysipelas and intertrigo (67).

Psychosocial complications include body dissatisfaction, symptoms of depression, loss-of-control in eating, unhealthy and extreme weight control behaviors, impaired social relationships and decreased health-related quality of life. These are conditions which show small to moderate associations with child and adolescent obesity. Complications with negligible to small associations include low self-esteem, clinically significant depression (of diagnostic severity, associated with significant distress and/or impairment, or inclusive of serious symptomatology), suicide attempts and full-syndrome eating disorders (69,70,71,72). Additional complications manifested in later life include decreased educational and financial attainment. As the medical setting is often the first point of contact for families, pediatricians are instrumental in the identification and referral of children with psychological complications (69,70,71,72). Motivational interviewing, patient talking points, brief screening measures and referral resources are important tools in this process.

**Clinical Approach to Overweight/Obese Children and Adolescents**

Calculation of BMI is a clinically practical tool for the assessment of overweight and obesity in children. All children older than two years should have their BMI calculated at least annually from measured height and weight. The results should be plotted on an appropriate growth curve. Use of weight, height and BMI growth charts not only as diagnostic tools but also as patient and family instruction/education is an instrumental approach in the author’s clinic.

The BMI percentile and trend of his/her percentile curve determine whether the child is underweight (<5th percentile), of normal weight (between 5th and 85th percentile), overweight (BMI ≥85th percentile and <95th percentile), or obese (≥95th percentile). Review of growth charts at each clinic visit is of utmost importance as this should help with early detection of an increasing BMI trend (more than three to four units -kg/m2- per year) and starting to cross percentile lines, indicating a need for early intervention (see section on treatment) (5).

**Clinical Evaluation**

**History**

The evaluation of the overweight/obese child should include a comprehensive history and physical examination. Laboratory and radiologic studies also may be obtained as indicated by the history and examination. The evaluation should also identify treatable causes and comorbidities (5,66). It is recommended to consider certain screening tests for a general metabolic assessment in all patients and pursue a more in-depth evaluation if and when indicated by the case-specific characteristics of the child being evaluated.

It is also noteworthy that the assessment of BMI must not be confined to the evaluation of dietary patterns and physical activity. Environmental and social supports and barriers, opinions on cause and effect of the problems and self-efficacy and readiness to change should be evaluated. It is the responsibility of the clinician to recognize the interactions between pediatric obesity and psychological complications and to engage patients and their caregivers accordingly. 

Patient history: Patient history should include the age of onset of overweight and information about the child’s eating and exercise habits. Age of onset is helpful in distinguishing overfeeding from genetic causes of overweight since syndromic obesity often has its onset before two years of age. It is also important to keep in mind the single gene defects associated with obesity which present with early-onset obesity (Table 2). Information obtained from the dietary and physical activity history may identify potential areas for intervention. A history of inability to control consumption of large amounts of food may be indicative of an eating disorder.

There is an association between skipping meals and overweight/obesity. In the author’s clinical practice, the most frequently encountered pattern is skipping breakfast. However, this is not a causal relationship (5,73). The medical history should include a review of all medications, particularly those that are known to be weight-promoting (antipsychotics such as thioridazine, risperidone and lithium carbonate; tricyclic antidepressants such as amitriptyline, antiepileptic drugs such as valproate, carbamezapine, gabapentin and hormones, especially corticosteroids and insulinotropic agents, insulin) (1,2,5,74).

The review of systems should search for evidence of comorbidities or underlying etiologies. For example, an abrupt onset of obesity with rapid weight gain should prompt investigation of medication-induced weight gain, a major psychosocial trigger (such as depression), endocrine causes of obesity (e.g., Cushing disease, hypothalamic tumor) or obesity syndromes such as Prader-Willi syndrome or rapid-onset obesity with hypothalamic dysfunction, hypoventilation, autonomic dysregulation and neural crest tumor (Rohhadnet syndrome) (5,75).

Family history: Obesity in one or both parents is an important predictor for whether a child’s obesity will persist into adulthood (76,77,78,79). The family history should include information about obesity in first-degree relatives (parents and siblings). It also should include information about common comorbidities of obesity, such as cardiovascular disease, hypertension, diabetes, liver or gallbladder disease and respiratory insufficiency in first and second-degree relatives (grandparents, uncles, aunts, half-siblings, nephews and nieces) (76,77,78,79).

Psychosocial history: The psychosocial history should include information related to depression, to school and social environment and to tobacco use (cigarette smoking increases the long-term cardiovascular risk).

The topics of weight and mental health issues must be approached with care and consideration (69). Evidence suggests that even health care professionals hold biased attitudes toward adult patients, biases that may be extended to younger patients (80). Biased attitudes may be manifested in a tendency to blame parents for their children’s weight status, negative comments on the parents’ or child’s weight or failure to listen to parents. Parents may feel guilt, anger and frustration because they do not know how to help their children, may feel they will be blamed for their children’s weight status or that their concerns and perspectives have been dismissed (81). Parents are instrumental in the provision of their children’s physical and social environments and a positive working relationship is essential to their children’s physical and mental health (69,81,82).

**Physical Examination**

The physical examination should evaluate the presence of comorbidities and underlying etiologies. Assessment of general appearance may help to distinguish the etiology of obesity. This assessment should include inspection for dysmorphic features which may suggest a genetic syndrome, assessment of affect and assessment of fat distribution. The excess fat in obesity resulting from overeating, i.e. exogenous obesity usually is distributed in the trunk and periphery. In contrast, the centripetal distribution of body fat (concentrated in the interscapular area, face, neck and trunk) is suggestive of Cushing syndrome. Abdominal obesity (also called central, visceral, android or male-type obesity) is associated with certain comorbidities, including the MetS, PCOS and insulin resistance. Measurement of the waist circumference, in conjunction with calculation of the BMI, may help to identify patients at risk for these comorbidities. Waist circumference standards for American children of various ethnic groups are available (81). There are numerous publications on waist circumference measurements in children from various geographic regions which may be utilized in individual clinics. 

Blood pressure should be measured carefully using a proper-sized cuff. The bladder of the cuff should cover at least 80 percent of the arm circumference (the width of the bladder will be about 40 percent of the arm circumference). In many obese children and adolescents, this will require the use of “adult” or “large adult” sized cuffs. Hypertension increases the long-term cardiovascular risk in overweight or obese children. In addition, hypertension may be a sign of Cushing syndrome (5,82). Hypertension is defined as a blood pressure greater than the 95th percentile for gender, age and height obtained on three separate occasions. Age- and height-specific blood pressure percentile references should be used (83).

Assessment of stature and height velocity is useful in distinguishing exogenous obesity from obesity that is secondary to genetic or endocrine abnormalities, including hypothalamic or pituitary lesions. Exogenous obesity drives linear height, so most obese children are tall for their age. In contrast, most endocrine and genetic causes of obesity are associated with short stature (5,66). Children with Prader-Willi syndrome are often short for their genetic potential and/or fail to have a pubertal growth spurt.

Examination of the head, eyes and throat may provide clues to the etiology of obesity and/or comorbidities (5,84). For example, microcephaly is a feature of Cohen syndrome. Blurred disc margins may indicate pseudotumor cerebri, an unexplained but not uncommon association with obesity (65,66). Nystagmus or visual complaints raise the possibility of a hypothalamic-pituitary lesion (85). Other findings that support this possibility are rapid onset of obesity or hyperphagia, decrease in growth velocity, precocious puberty or neurologic symptoms. Clumps of pigment in the peripheral retina may indicate retinitis pigmentosa, which occurs in Bardet-Biedl syndrome (5,86). Enlarged tonsils may indicate obstructive sleep apnea. Erosion of the tooth enamel may indicate self-induced vomiting in patients with an eating disorder.

Dry, coarse or brittle hair may be present in hypothyroidism. Striae and ecchymoses may be manifestations of Cushing syndrome; however, striae are much more likely to be the result of rapid accumulation of subcutaneous fat. Acanthosis nigricans may signify T2DM or insulin resistance. Hirsutism may be present in PCOS and Cushing syndrome (67,68,84).

Abdominal tenderness may be a sign of gallbladder disease. Hepatomegaly may be a clue to NAFLD (9,84).

The musculoskeletal examination may provide evidence of underlying etiology or comorbidity of childhood overweight. Nonpitting edema may indicate hypothyroidism. Postaxial polydactyly (an extra digit next to the fifth digit) may be present in Bardet-Biedl syndrome and small hands and feet may be present in Prader-Willi syndrome (5,86). The musculoskeletal examination may provide evidence of SCFE (limited range of motion at the hip, gait abnormality) or Blount disease (bowing of the lower legs). Dorsal finger callousness may be a clue to self-induced vomiting in patients with an eating disorder (82). 

The genitourinary examination and evaluation of pubertal stage may provide evidence for genetic or endocrine causes of obesity (82). Undescended testicles, small penis and scrotal hypoplasia may indicate Prader-Willi syndrome. Small testes may suggest Prader-Willi or Bardet-Biedl syndrome (5,86). Delayed or absent puberty may occur in the presence of hypothalamic-pituitary tumors, Prader-Willi syndrome, Bardet-Biedl syndrome, leptin deficiency or leptin receptor deficiency (5,10,86). Precocious puberty occasionally is a presenting symptom of a hypothalamic-pituitary lesion (85).

Most of the syndromic causes of overweight in children listed in Table 2 are associated with cognitive or developmental delay. Prader-Willi syndrome is also associated with marked hypotonia during infancy and delayed development of gross motor skills. 

**Laboratory Studies**

The laboratory evaluation for overweight and obesity in children is not fully standardized. Most clinicians use a basic panel of tests to screen for T2DM, dyslipidemia and fatty liver disease in children with BMI values ≥85th or ≥95th percentiles. In the author’s clinic, the initial clinical evaluation is followed by a comprehensive metabolic profile, thyroid screening (free T4 and TSH) and hemoglobin A1c (HbA1c) level. The child is likely to be non-fasting at the time of initial clinical evaluation. The metabolic panel provides a random glucose, serum alanine aminotransferase (ALT) and aspartate aminotransferase. HbA1c is a useful marker of the average blood glucose concentration over the preceding 8 to 12 weeks. Because of improved assay standardization and validation against other diagnostic methods, HbA1c has recently gained more emphasis as a screening tool for diabetes mellitus. In year 2010, the American Diabetes Association (ADA) authorized the use of HbA1c as a diagnostic criterion for diabetes and other glucose abnormalities provided that an assay that is certified by the National Glycohemoglobin Standardization Program is used (87). The prediabetic state was defined as HbA1c ≥5.7% and <6.5% in adults. The primary potential benefit of using HbA1c is practicality, i.e. the patient does not need to be fasting and testing does not require a return visit to the physician or the laboratory. Furthermore, HbA1c has less variability in repeat studies compared with fasting glucose values. HbA1c is also less subject to acute changes such as illness-/stress-induced hyperglycemia and short-term exposure to medications that induce hyperglycemia. However, there are also some disadvantages of use of HbA1c for the screening of diabetes in the pediatric population. For example, diseases such as iron-deficiency anemia, cystic fibrosis, sickle-cell disease, thalassemia and other hemoglobinopathies alter HbA1c results. Ethnic variation in HbA1c levels has also been reported. The high cost of the test is also a handicap. Therefore, clinical judgment should be used (88). Recent studies have shown that health care providers started to include HbA1c in their screening practices and are more willing to include this test in the context of the recent ADA guidelines (89).

The next laboratory evaluation in the author’s clinic is scheduled as a fasting blood draw which is mostly coordinated with a nutrition/coordinator nurse consult for convenience purposes. The fasting laboratory tests include a lipid panel (total cholesterol, triglycerides, LDL cholesterol and HDL cholesterol), fasting glucose and insulin. A fasting insulin level may be included for purposes of counseling rather than screening, as also recommended by an international consensus report on pediatric insulin resistance in 2010 (90). 

Based on the ADA Consensus Panel Guidelines from year 2000, screening for diabetes should be performed in children over 10 years of age (or at the onset of puberty if it occurs at a younger age) who are overweight or obese and have two or more additional risk factors. The additional risk factors include a family history of T2DM in a first- or second-degree relative, high-risk ethnicity, acanthosis nigricans or PCOS (91). The ADA recommends measurement of fasting plasma glucose level in these patients. In the context of the new screening guidelines advocating the use of HbA1c, clinical approaches are likely to incorporate this test.

In the author’s clinic, children and adolescents who display multiple risk markers for dysglycemia (prediabetes or diabetes) undergo a basic two-hour oral glucose tolerance test (OGTT) with fasting and 2-hour blood samples for glucose and insulin. The risk markers consist of strong family history of T2DM or intrauterine exposure to diabetes (maternal gestational DM), clinical indications for insulin resistance (acanthosis nigricans, PCOS) and a HbA1c level in the suspicious zone for prediabetes (5.7%-6.4% per ADA guidelines). Clinical judgment is also a key in making these decisions. A fasting glucose of 100 to 125 mg/dL (5.55 to 6.94 mmol/L) is considered to be prediabetic (impaired fasting glucose) and a level of ≥126 mg per dL (7.0 mmol/L) is consistent with the diagnosis of diabetes. Children with an elevated fasting glucose should have a confirmatory OGTT. Patients with intermediate or conflicting results for any of these tests should undergo repeat testing and be monitored for future development of diabetes. Definitive diagnosis of diabetes mellitus requires meeting diagnostic criteria on at least two separate occasions (87).

In 2011, an expert panel from the US National Heart, Lung and Blood Institute (NHLBI) issued integrated guidelines for cardiovascular risk reduction, endorsed by the American Academy of Pediatrics (92). The panel recommended initial lipid screening with fasting lipid profile for children between two and eight years of age with a BMI ≥95th percentile (or other risk factors for cardiovascular disease, such as family history of dyslipidemia/early cardiovascular disease and/or morbidity in first- or second-degree relatives, history of diabetes, hypertension, or smoking in childhood). The panel recommended universal lipid screening with a non-fasting non-HDL cholesterol (subtracting the HDL from the total cholesterol measurement) for children of ages 9-11 years and 17-21 years. For children of ages 9 and above with a BMI ≥85th percentile or other risk factors as listed above, lipid screening is recommended (92). Thresholds for normal are a fasting total cholesterol of <170 mg/dL, a LDL cholesterol of <110 mg/dL, non-HDL cholesterol of <120 mg/dL, triglycerides of <75 mg/dL for ages 0-9 years and <90 mg/dL for ages 10-19 years and HDL cholesterol of >45 mg/dL. Abnormal values compatible with dyslipidemia are a fasting total cholesterol of ≥200 mg/dL, a LDL cholesterol of ≥130 mg/dL, non-HDL cholesterol of ≥145 mg/dL, triglycerides of ≥100 mg/dL for ages 0-9 and ≥130 mg/dL for ages 10-19 years and a HDL cholesterol of <40 mg/dL. Overweight/obese children with hyperlipidemia should be monitored and treated. Stepwise approach includes lifestyle modification and medical therapy if indicated (92,93,94). In broad terms, two forms of approach are recommended for the management of hyperlipidemia. The first is a population-based approach to improve lifestyle and lipid levels in all children. The second is a high-risk strategy to identify children with genetic and environmental dyslipidemias by screening and treating as indicated (92,93,94).

Liver function tests should be obtained because NAFLD is typically asymptomatic (5,9,59). Obese children with an elevation of ALT greater than two times the norm that persists for greater than three months should be evaluated for the presence of NAFLD and other chronic liver diseases (e.g., viral hepatitis, autoimmune hepatitis, Wilson disease, a1- antitrypsin deficiency). Assessment for other comorbidities including sleep apnea and PCOS depends on the presence of risk factors or symptoms and should be pursued on a case-by-case basis if indicated. Vitamin D deficiency appears to be common among children and adolescents with obesity either because of generalized vitamin and mineral deficiencies secondary to poor eating habits and/or due to sequestration in excess adipose tissue. Studies from various geographical regions reported that vitamin D deficiency was present in about half of children and adults with severe obesity and was associated with higher BMI and features of the MetS (95,96,97,98). There are no guidelines recommending routine screening of overweight children for vitamin D status, therefore, clinical judgment is recommended. If screening for vitamin D deficiency is undertaken, levels are measured as serum 25 hydroxyvitamin D. The reference range varies by region, but levels <20 ng/mL are generally considered as deficient. In populations of children with obesity, vitamin D deficiency was not generally associated with overt clinical symptoms (95,96,97,98). However, if deficiency is found, vitamin D supplementation should be initiated to avoid long-term consequences.

Additional testing should be performed as needed if there are findings consistent with hypothyroidism, PCOS, Cushing syndrome and sleep apnea (5,39,66,99). Syndromic obesity should be evaluated in children with developmental delay or dysmorphic features. As previously discussed, endocrine causes of obesity are unlikely if the growth velocity is normal during childhood or early adolescence (66). 

Radiographic evaluation of overweight or obese children may be pursued if indicated by findings in the history and physical examination. For example, plain radiographs of the lower extremities should be obtained if there are clinical findings consistent with SCFE (hip or knee pain, limited range of motion, abnormal gait) or Blount disease (bowed tibia). Abdominal ultrasonography may be indicated in children with findings consistent with gallstones (e.g., abdominal pain, abnormal transaminases) (5). Abdominal ultrasonography may be used to confirm the presence of fatty liver.

**Treatment**

Pharmacotherapy options for the treatment of pediatric obesity are very limited. Therefore, it is crucial to establish a comprehensive management program that emphasizes appropriate nutrition, exercise and behavior modification.

Lifestyle modification involving nutrition and physical activity (i.e. non-pharmacologic treatment) remains as the foundation of the treatment approach to childhood obesity. It is crucial to involve the family/caregivers of the child in this approach. Behavioral change is needed to improve the energy balance, i.e. to reverse the disproportionately high energy intake compared with energy expenditure.

Treatment should encompass the concepts of primary and secondary prevention. Primary prevention is targeted at the prevention of overweight/obesity, whereas, secondary prevention should target prevention of complications/comorbidities. In either case, a family-based approach will help extend these concepts of prevention to the other family members.

Treatment/prevention efforts should start with the detection of an increasing BMI trend. If the increase in the BMI is more than three to four units (kg/m2) per year and starting to cross percentile lines, even when the BMI is still below the 85th percentile (particularly if the child is older than four years), this should prompt the clinician to discuss these observations with the family (5). Providing simple tips for maintaining a healthy weight by nutrition modification and increased physical activity and parenting strategies to support these goals will be a good start. 

If the child is overweight (the BMI is ≥85th percentile but less than the 95th percentile), or obese (the BMI is ≥95th percentile), he or she should be screened for comorbidities of obesity. The clinician should provide counseling to optimize lifestyle habits with a goal of slowing the rate of weight gain. These families should be provided with information about a healthy lifestyle and direct counseling from a dietitian to address their specific challenges should be considered. These children, particularly the obese ones, will require regular follow-up to monitor progress. It is also important to note that the term “obesity” is an emotionally charged word for most children and the author prefers to use the terms overweight and at risk of overweight in clinical practice.

The 2011 NHLBI Expert Panel integrated guidelines for cardiovascular health and risk reduction in children and adolescents is a comprehensive reference prepared to assist pediatric health care providers in both the promotion of cardiovascular health and the identification and management of specific risk factors (92). Among the risk factors, obesity plays a central role as obesity tracks more strongly than any other risk factor from childhood into adult life. The reader is referred to this reference as a comprehensive guide for clinical management of overweight/obese children (92). It is of utmost importance to have a stepwise approach in goal setting. Primordial prevention necessitates the prevention of risk-factor development. Primary prevention encompasses effective management of identified risk factors for the prevention of future problems such as cardiovascular disease, prediabetes, diabetes and other potential complications.

Children who have comorbidities of obesity should be referred to appropriate subspecialty services. Based on availability, children may be referred to pediatric obesity centers for appropriate dietary, pharmacologic and/or surgical therapy.

Treatment of obesity must help achieve a negative energy balance (100). The preferred route of accomplishing this is through reducing caloric intake and increasing energy expenditure. This strategy is in congruence with the first law of thermodynamics. Dietary intervention (nutrition therapy) is the mainstay of the reduction of caloric intake and the insulin response that promotes excessive energy deposition into adipose tissue (31). Reduced-energy diets regardless of macronutrient composition result with clinically meaningful weight loss in adults. Available randomized control trial data on dietary intervention in youth are relatively sparse, partly explainable by the high attrition rates and relatively short-term follow-up periods (31). The association between pediatric obesity and the consumption of high-calorie high-fat high-carbohydrate low-fiber foods has been demonstrated by multiple studies (101). The specific approaches which have helped reducing obesity in children include elimination of sugar-containing beverages and a transition to low-glycemic index diet (31). It is crucial to increase awareness of the families of the caloric content of the beverages, in particular the sugar-containing beverages. The next step is education of the family to limit the intake of sugary beverages. In the author’s clinical experience, it is unexpectedly common that most parents would regard natural fruit juices as “guilt-free” and provide unlimited amounts to their children unless counseled.

Physical activity intervention is the other mainstay of obesity treatment. A recent systematic review and meta-analysis concluded that both diet-only and diet plus exercise interventions resulted in weight loss and metabolic profile improvement (102). However, it was noted that the addition of exercise to dietary intervention led to greater improvements in HDL cholesterol, fasting glucose and fasting insulin levels (102).

Behavior modification as an approach to weight loss may include motivation to reduce screen time and increase physical activity, psychologic training to accomplish a change in eating behaviors or exercise, family counseling to support weight loss goals, and school-based changes to promote physical activity and healthy eating (17). Although several forms of medications to treat obesity are on the market, only one is approved for children aged less than 16 years. Success has been limited with these medications and the current understanding is that these may only be used as an adjunct to exercise and nutrition interventions. Due to the detection of serious adverse effects, the majority of the medications proposed to treat obesity have been removed from use in the pediatric age group. A thorough discussion of the pharmacotherapy targeted at pediatric weight management is beyond the scope of this paper. Therefore, a few will be briefly summarized and the reader will be referred to specific reviews (17,103). Pharmacologic treatment may be considered in selected subjects, especially in the presence of significant and severe comorbidities, when lifestyle intervention has failed to achieve weight reduction. Orlistat (appetite suppressor) and sibutramine (gastrointestinal lipase inhibitor) are FDA-approved for treatment of pediatric obesity. Metformin may be considered in the presence of clinically significant insulin resistance. Evidence is lacking on the appropriate duration of medical therapy and its optimal combination with lifestyle intervention. Lack of coverage of medications by insurance and high out-of-pocket costs may be limiting factors to some families. Adverse effects necessitate careful monitoring and may lead to discontinuation of medication. Therapies altering insulin resistance, in particular metformin, are currently approved for treatment of T2DM in children aged 10 years and older. Several randomized controlled trials have evaluated metformin as an “obesity medication” in adolescents. The extents of the long-term effect of metformin on body weight or its complications are unknown. Leptin has been shown to be effective in reducing BMI among individuals with true leptin deficiency, which is a rare condition (104). Octreotide has been used in the treatment of hypothalamic obesity and shown to suppress insulin and stabilize weight and BMI (2,105). Bariatric surgery is the most definitive and longest lasting form of weight loss treatment. In adults and to a smaller extent in adolescents, bariatric surgery has been shown to result in significant weight loss and improvement or resolution of multiple comorbidities, such as T2DM, hypertension and obstructive sleep apnea. However, there are significant acute and chronic complications of this surgery, ranging from wound infections to life-threatening complications such as bowel obstruction/perforation (2,4,17,106).

Approach to comorbid conditions with appropriate treatment strategies is essential. For example hormonal contraceptives are the first-line management for menstrual abnormalities and hirsutism/acne in PCOS. Hormonal contraceptives and metformin are the treatment options in adolescents with PCOS.

Hypertension may necessitate medical treatment.

Over the past 2 decades, several pediatrics clinics have shown concentrated efforts to initiate specialty clinics focusing on the management of overweight/obese children and adolescents. Some of these clinics have been supported by research/clinic support grants. It may neither be realistic nor feasible to establish a similar center in each institution as this is highly contingent upon staffing and funding. Therefore, other solutions need to be developed. In pediatric clinical centers, training of the health care personnel on evaluation and management of pediatric overweight/obesity should be the first step to incorporate a structured and comprehensive evaluation into the routine clinic practice. This should enable identification of the comorbidities and subspecialty referrals needed. Our pediatric endocrinology division has recently become part of a collaborative obesity-prevention project entitled Healthy Green and Into the Outdoors organized by the Community Foundation of North Louisiana, supported by a grant from Blue Cross Blue Shield Foundation of Louisiana. Currently, in a busy clinical setting of a tertiary academic referral center, we are developing a model where the resources of general pediatrics and pediatric endocrinology sections are combined to accomplish goals. The model also involves availability of a coordinator nurse who serves as a lifestyle counselor for the children and families. 

At present, there is a crucial need for pediatric obesity advocacy. This necessitates the clinician’s involvement beyond the office, collaboration with the community and public policy makers. The magnitude of the current pediatric obesity problem renders it necessary to maximize preventative efforts. Clinicians are called to intervene on behalf of their parents not only in the clinical setting but in the community as well (9). Indeed, the 2005 Institute of Medicine Report recommends that clinicians, regardless of specialty, serve as role models and provide leadership in their communities for obesity prevention efforts (107). Pediatricians, regardless of their subspecialty, should consider routinely discussing obesity prevention and recommendations with patients and families (9). Clinicians should consider advocating for and providing healthy food options in hospitals. In various areas of the US, successful models have been established involving the medical/hospital staff in community advocacy programs, community/hospital partnerships, locally and at state level. Physician participation may involve speaking at public forums and attending community/school board meetings to offer medical expertise on the issue of pediatric obesity. The American Board of Pediatrics maintenance of board certification (MOC) programs now involve several projects focusing on pediatric obesity and its management. These educational modules help with knowledge acquisition, self-efficacy and physician compliance with certain practice recommendations for the screening, prevention and management of pediatric obesity and provide credit for MOC (108). 

**Conclusion/Future**

Pediatric care providers should universally assess children for obesity risk to improve early identification of elevated BMI, medical risks and unhealthy eating and physical activity habits. This should start in the primary care setting. Calculation of the BMI and plotting on an appropriate reference curve should be part of the routine clinical assessment of all children of ages 2 years and above. Measured heights and weights should be used for accuracy. The sequential follow-up for the trends may provide invaluable and timely information to track overweight/obesity trends and may be instrumental to initiate primary and/or secondary prevention (5). It is important to note that electronic health record programs may be instrumental in calculation, plotting and follow-up of the BMI and pertinent anthropometric measures and increase clinical efficacy. Providers can provide obesity prevention messages for most children and suggest weight-control interventions for those with excess weight (109). As pediatric health care providers, we have always believed that it is of utmost importance to recall the statement “children are not just small adults” during clinical evaluation and treatment

## Figures and Tables

**Table 1:**
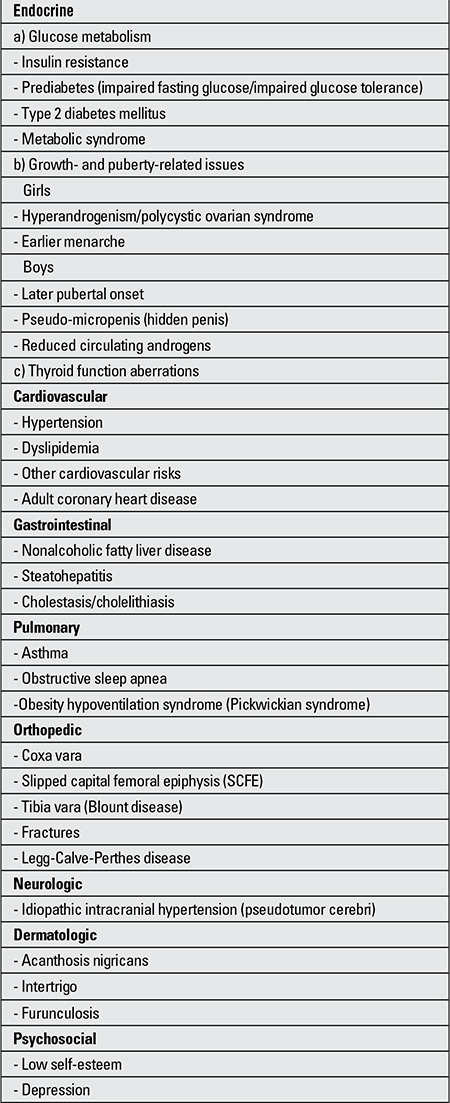
Comorbidities and complications of childhood obesity

**Table 2:**
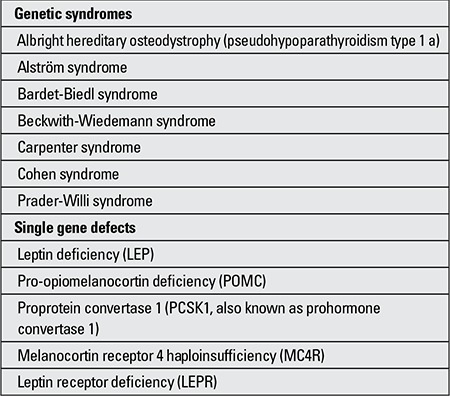
Genetic syndromes and associated with obesity (modifiedfrom Klish WJ) (5)
